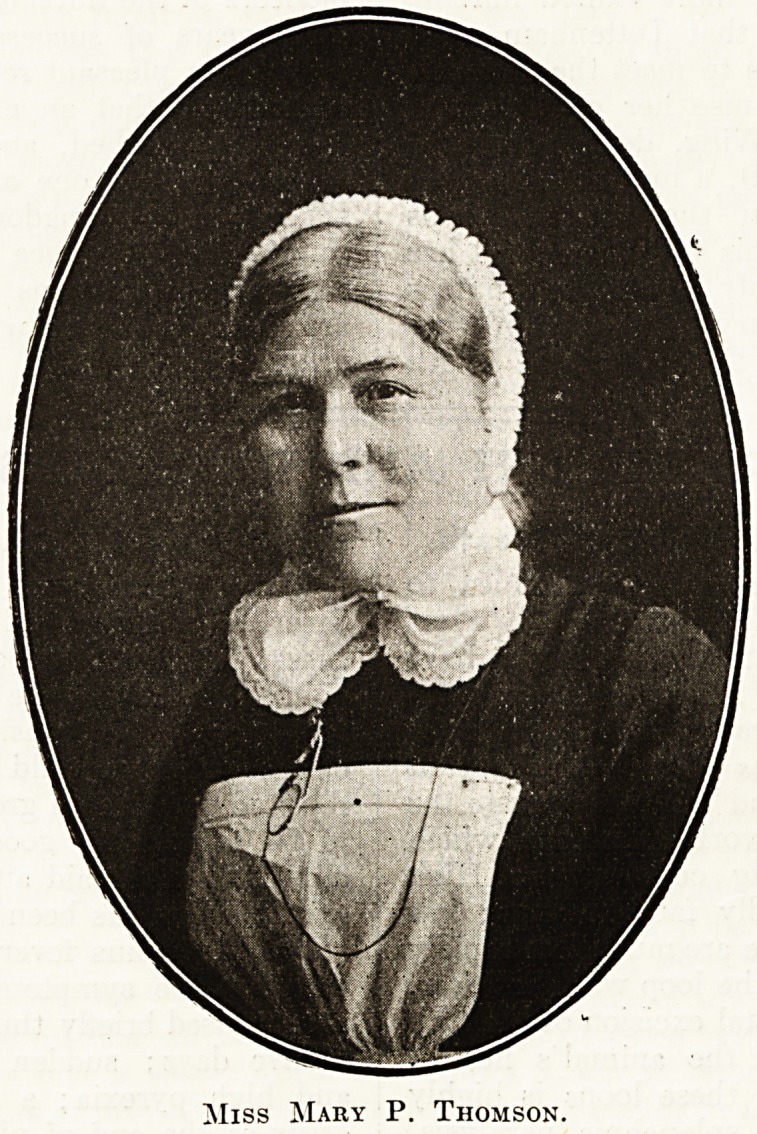# Sister Mary (Miss Mary P. Thomson), for Forty Years Lady Superintendent, Royal Infirmary, Sunderland

**Published:** 1912-08-24

**Authors:** 


### WOMEN BEHIND THE HOSPITALS.

### Sister Mary (Miss Mary P. Thomson), for Forty Years Lady Superintendent, Royal Infirmary, Sunderland.

Miss Thomson, better known as "Sister Mary," has just resigned the office of Lady Superintendent at the Royal Infirmary, Sunderland, where she has worked for forty years. In his Report on this institution published in The Hospital on March 23 of the present year, page 635, Sir Henry Burdett gave an account of the present condition of this institution. He recorded that Miss Thomson commenced her career at the Evangelical Protestant Deaconess' Hospital and Training Institution, Tottenham, which institution has long since been merged into and absorbed by the Prince of Wales' General Hospital, Tottenham.

For many years Sister Mary discharged the duties of Lady Superintendent at Sunderland in an honorary capacity, but some years ago, after the Committee had decided to employ no unpaid labour for the future, she consented to the Committee's wish that she should be paid a salary. Sister Mary's work has been remarkable in the sense of the influence she has exercised by her commanding personality during the long period while she held control at Sunderland Infirmary. Some idea of her character and gifts may be obtained from the excellent likeness we are privileged to give in the present number. We are indebted to Dr. Morgan, chairman of the medical board, and to Miss J. R. Armour for the following account of her connection with nursing and of her work. Miss Armour has succeeded Sister Mary as Superintendent at the Royal Infirmary, Sunderland, after many years' excellent work as assistant matron, during which period she gained a complete knowledge of the whole institution and its requirements. She is a qualified and worthy successor to Sister Mary, and we have little doubt that under her control the institution will progress, and that the long-needed reconstruction and provision of adequate accommodation for the staff will speedily be taken in hand:—

When we see the well-equipped hospitals of to-day it is well to remember what they were fifty years ago, when the nurse was an untaught, untrained, and often unwashed servant-girl. The pioneers who led the way to our present improved position are worthy of all honour. We are all familiar with the heroic work of Miss Florence Nightingale, and many other noble efforts prompted by her example have lifted us to a higher level. It was a great and noble conception which led to this work at Kaiserswerth and, what is sometimes forgotten, to the foundation of the daughter-institution which Dr. Laseron founded at Tottenham. There earnest women were led to consecrate themselves to the service of God by acquiring the knowledge and methods of relieving sickness. In these early days, it is true, systematic teaching was not sufficiently enforced. Yet, by and by, systematic training came, while the religious tone still continues to ennoble the work.

Miss Mary P. Thomson.

In the year 1872 the Committee of the Sunderland Infirmary, having moved into their new hospital, and having left the wretched, ill-conditioned house where for many years the treatment of surgical cases, of medical cases, and of infectious diseases was carried on promiscuously and under one roof, became convinced that some approved system of nursing should take the place of the very casual nursing which was then all that could be depended upon. They sent a deputation to London to ascertain what was being done there and to recommend what should be done in their own institution. That deputation visited and inspected several training schools, and recommended that Dr. Laseron's kind offer to undertake the nursing of the Sunderland Infirmary should be accepted.

In the spring of 1873 Sister Mary Paterson Thomson left her home in Inverness and entered Tottenham Hospital, now called the Prince of Wales' Hospital, as a probationary deaconess. On June 9 in the same year she was one of the little band of seven sisters sent at the request of the board of management to undertake the nursing and organisation of the Sunderland Infirmary. In 1875 she returned to Tottenham to be "set apart" as a deaconess, and to receive some months of special training. Sister Mary returned to Sunderland in November of that year. There were then but 100 beds, and these seven ladies bravely took charge of them. They had no restricted rules as to hours and class of work. The work had to be done and they did it. They polished the stoves, scrubbed the floors; they kept the patients fed and clean and comfortable; they attended in the operation theatre; and they dispensed the medicines. This questionable system had its use in consolidating the sisterhood in discipline and obedience, and it certainly has produced splendid results in the character and lives of those who were so tried.

The seven sisters chose as their head Sister Mary, and by and by their number rose to over twenty, while the work of the Infirmary grew in bulk and became more and more perfect in the detail of nursing, as wardmaids were engaged to do the menial work. From 1873 until 1889, during which time a large surgical wing, the backhouse, and an out-patient department had been added, the Infirmary was staffed entirely by deaconesses. But Sister Mary found that more skilled nursing was indispensable, and also that Tottenham could not continue to supply sisters to meet the demand. She felt that she must organise her own supply, and so, when the Hartley Wing, designed for 70 children, was opened in 1889, a number of nurses were received and placed in the various wards throughout the hospital. This training school has been most successful, not only in providing nurses for the Sunderland Infirmary, but in supplying skilled and competent nurses throughout the length and breadth of the kingdom. So much so, indeed, that to-day nurses trained in the Sunderland Infirmary are the valued heads of many of the institutions both at home and abroad. As the sisters have retired from time to time their places have been filled by charge nurses trained in the institution. An isolation block and nurses' home, also a Convalescent Home at Harrogate accommodating forty patients, have been added to the Infirmary—equipped and supervised by Sister Mary.

In 1900 Sister Mary, hoping to create a greater interest in the Infirmary among the ladies of Sunderland, proposed and organised a "Ladies Guild," consisting of thirty captains and three hundred members. During the eleven ensuing years this Guild has succeeded in adding to the endowment fund nearly £5,000. Sister Mary, one of the pioneers of the nursing profession, on whom nearly forty years of successful and devoted work have only left a pleasant retrospect, has the satisfaction of knowing that an excellent standard of training has been reached, and that the Royal Infirmary is recognised as one of the best provincial training schools in the kingdom. In this connection it is interesting to notice that at the present time eighteen matronships and two superintendentships are filled by nurses who have been trained in this school.

## Figures and Tables

**Figure f1:**